# Somatic *MED12* Mutations in Myometrial Cells

**DOI:** 10.3390/cells13171432

**Published:** 2024-08-27

**Authors:** Yinuo Li, Huma Asif, Yue Feng, Julie J. Kim, Jian-Jun Wei

**Affiliations:** 1 Department of Pathology, Northwestern University, Chicago, IL 60611, USA; sd09lyn@163.com (Y.L.); yue-feng@northwestern.edu (Y.F.); 2 Department of Obstetrics and Gynecology, Northwestern University, Chicago, IL 60611, USA; huma.asif@northwestern.edu

**Keywords:** myometrium, duplex deep sequencing, MED12 mutation, leiomyoma

## Abstract

Over 70% of leiomyoma (LM) harbor *MED12* mutations, primarily in exon 2 at c.130-131 (GG). Myometrial cells are the cell origin of leiomyoma, but the MED12 mutation status in non-neoplastic myometrial cells is unknown. In this study, we investigated the mutation burden of *MED12* in myometrium. As traditional Sanger or even NGS sequencing may not be able to detect *MED12* mutations that are lower than 0.1% in the testing sample, we used duplex deep sequencing analysis (DDS) to overcome this limitation. Tumor-free myometria (confirmed by pathology evaluation) were dissected, and genomic DNA from *MED12* exon 2 (test) and TP53 exon 5 (control) were captured by customer-designed probe sets, followed by DDS. Notably, DDS demonstrated that myometrial cells harbored a high frequency of mutations in *MED12* exon 2 and predominantly in code c.130-131. In contrast, the baseline mutations in other coding sequences of *MED12* exon 2 as well as in the TP53 mutation hotspot, c.477-488 were comparably low in myometrial cells. This is the first report demonstrating a non-random accumulation of *MED12* mutations at c.130-131 sites in non-neoplastic myometrial cells which provide molecular evidence of early somatic mutation events in myometrial cells. This early mutation may contribute to the cell origin for uterine LM development in women of reproductive age.

## 1. Introduction

Uterine leiomyomas (LM) occur in up to 77% of women of reproductive age [1,2]. It was until recently that the major driving genes for LM were discovered by whole genomic sequencing analysis. *MED12* (mediator complex subunit 12) is among the most common mutations found in >70% of uterine LM [3,4]. MED12 encodes a large gene with a total of 45 exons and *MED12* mutations in LM are exclusively confined in exon 2 and the adjacent intron 1–exon 2 boundary. The dominant hotspots are at codon 44 c.130G-131G [3]. *MED12* mutations are correlated with the number of LM in the uterus [5,6]. *MED12* mutations were found only in a few mesenchymal tumors, including LM and fibroadenoma of the breast [7], suggesting the selective mechanisms of MED12 in tumorigenesis of certain cell types.

To date, the molecular cause of such prevalent *MED12* mutations remains unknown. Previously, we have demonstrated that *MED12* mutations (predominantly at c.130-131 of exon 2) could be induced in myometrial cells with chronic exposure to reactive oxygen species (ROS) in vitro, suggesting that a mutation in this region could be an early event for the initiation of LM development [8]. The uterus is exposed to high levels of reactive oxidative species (ROS), mediated by cycle steroid hormone changes, hypoxic environment, and ROS-related tissue functions [9,10,11,12]. Moreover, ROS is a potent carcinogenic factor that induces DNA damage and mutations [13,14], with the accumulation of oxidative nucleotides in genomic DNA [15,16], dominantly guanine 8-OHdG (8-hydroxy-2′–deoxyguanosine). A high ROS burden promotes 8-OHdG formation in nuclei [15,16]. 8-OHdG causes misrepair and results in G transversions. This misrepair will allow 8-OHdG repairing with dATP and produces a stable G to T transversion, which is the hallmark of oxidative mutagenesis [13,17]. Detection of 8-OHdG misrepair induced by ROS has been tested at specific genes as well as globally [18] such as those found for *K-Ras* and *p53* [13,14]. Thus the association of ROS, DNA damage, and *MED12* mutations needs further investigation.

It is unclear when mutations occur in LM or whether non-neoplastic myometrial cells in reproductive-aged women gain somatic gene mutations, particularly in the *MED12* gene. Unfortunately, in order to detect mutations in a small subset of myometrial cells, conventional next-generation sequencing is insufficient in sensitivity as it is unable to detect the mutations at a frequency of <1% and almost impossible at <0.1% [19]. Deep sequencing analysis is a useful technique that can be used to detect extremely low-frequency mutations of individual cells in a large non-mutant cell population [20]. However, deep sequencing may yield a false positive error rate due to multiple steps of DNA preparation, PCR amplification, and sequencing [21]. The development of duplex deep sequencing (DDS) has enabled superior sensitivity and accuracy as it simultaneously sequences both strands of DNA and avoid errors inherent of the procedure [22,23].

In this study, we investigate *MED12* mutations in normal myometrial cells from hysterectomy specimens as an early genetic mutational event using DDS. For comparison purposes, *TP53* exon 5 was sequenced to understand the overall misrepair/mutation burden in the myometrium.

## 2. Materials and Methods

### 2.1. Study Design and Case Selection

Uterine tissues were collected from pre- and peri-menopausal women with hysterectomy at Northwestern University Prentice Women’s Hospital. Fresh tissues of LM and matched tumor-free myometrium from a total of 32 women were collected after informed consent. Histologic evaluation was performed by the pathologist to ensure the myometrium was absent of microscopic LM tumors. The dominant LM was also collected for *MED12* mutation analysis. Patient age at surgery, race, uterine weight, and number of tumors were documented in Table 1. This study obtained approval from the Northwestern Institutional Review Board (IRB) (STU00212471).

### 2.2. Histologic Evaluation Criteria

Fresh tissue samples from an area of tumor-free myometrium were bisected. One-half of the tissue sample was snap-frozen for molecular testing, and the other half was processed for histologic evaluation. Routine hematoxylin and eosin-stained slides were prepared from formalin-fixed and paraffin-embedded (FFPE) tissue samples to ensure no LM contamination. All selected myometrial tissue samples for this study were LM-free at the microscopic level. The dominant LM from each case were also collected for histology evaluations, and they were all usual type LM.

### 2.3. DNA Extraction and Sanger Sequencing

DNA extraction was performed in fresh/frozen and FFPE tissues with aid of ZYMO RESEARCH Quick-DNA™ MiniPrep Plus Kit and ZYMO RESEARCH Quick-DNA™ FFPE Kit (ZYMO RESEARCH, D4068, D3067, lrvine, CA, USA), respectively, following the manufacturer’s instruction. In brief, 10 µm FFPE tissue sections were collected into 1.5 mL Eppendorf tubes and deparaffinized. For fresh/frozen tissues, grinded tissue were digested with a proteinase K at 56 °C for 1–4 h followed by incubation at 90 °C for 20 min. DNA was purified by column purification and was quantitated using NanoDrop Spectrophotometer (Thermo fisher, Waltham, MA, USA) and DNA quality was further assessed by A260/A280 ratio and its integrity by 1% agarose gel electrophoresis. For the Sanger sequencing, *MED12* exon 2 was amplified by PCR with 50 ng of genomic DNA with primers 5′-ACAACTAAACGCCGCTTTCCT-3′ (forward) and 5′-GGGCCTTTGCTCCTTCTTAG-3′ (reverse). The purified PCR products by ExoSAP-IT (Affymetrix, Inc.) were sent to NU sequence core for Sanger DNA sequencing with Applied Biosystem’s 3730xl DNA Analyzer (Torrance, CA, USA). Utations/variations were analyzed by software FinchTV 1.5 and Indigo (https://www.gear-genomics.com/indigo/).

#### DNA Library Preparation and Duplex Deep Sequencing

Genomic DNA from myometrium was prepared by the ZYMO RESEARCH Quick-DNA™ MiniPrep Plus Kit. In preparation for duplex sequencing [22,23], DNA probe sets for MED12 and p53 were purchased from IDT, which covered the entire target sequences for the regions of interest and captured *MED12* exon 2 and *P53* exon 5. The genomic DNA was sonicated, end-repaired, A-tailed, and ligated with UMI (IDT) and index adapters (IDT) using the KAPA HyperPrep library kit (Roche Sequencing, Basel, Switzerland). The DNA quality is shown in Figure S1. After genomic DNA were amplified by 5 PCR cycles, the biotinylated oligonucleotide probes (IDT) were used to capture the DNA fragments from *MED12* exon2, *p53* exon 5 with xGen^®^ Lockdown^®^ Reagents (IDT) following the manufacturer’s instruction. After two rounds of target sequence captures, the sufficient target enrichment will be obtained. Then, the libraries of capture DNA fragments were prepared and proceeded to sequenced in 2 × 150 paired-end reads with eight-base indexing read on an Illumina NovaSeq 6000 (Illumina, San Diego, CA, USA).

For duplex sequencing, data generated from deep sequencing were processed based on the published method [23]. Briefly, FASTQ files were converted to unaligned BAM using Picard FastqToSam. Reads were grouped based on the unique tagged sequences to generate read families needed to make single-strand consensus sequences (SSCS) and duplex consensus sequences (DCS) using Unified Consensus Maker (UCM). Consensus sequences were aligned to the reference hg38 genome using Burrows-Wheeler aligner (BWA-mem) and variants calling was performed using VarDict (Java). The sequence was assembled into an error-corrected Duplex Consensus Sequence by Samtools-Mpileup and GATK4 (detail methods described [24]). Double-stranded molecular barcodes, which used to identify the specific sequence and reads derived from both strands of DNA molecule. Mutations were recorded when they were detected in both DNA strands. This would eliminate any errors that occurred in single strand. *MED12* and *p53* of each nucleotide mutation frequencies were calculated according to the identified mutations divided by the total number of Duplex nucleotides sequenced. The mutant allele frequency (MAF) was calculated based on the mutated Duplex bases divided by the total DS reads at a given nucleotide position [25].

### 2.4. Statistical Analysis

Statistical analysis was performed using GraphPad Prism software v9 (GraphPad Software). An unpaired *t*-test was applied to compare two groups. A paired t-test was applied for matched data from two groups. When necessary, the ordinary one-way ANOVA, Brown–Forsythe, and Welch ANOVA, or Kruskal–Wallis tests might be used for multiple group comparisons depending upon the distribution and variances of the data set. The mean ± SEM from three or more independent experiments were calculated. Data or comparison with *p*-value < 0.05 were considered statistically significant.

## 3. Results

### 3.1. Case Selection and Sanger Sequencing Analysis of MED12 in LM

A total of 32 cases for LM, including 16 black and 16 white age-matched women, from hysterectomies, were selected for this study. Patients’ ages ranged from 27 to 55 years old. Overall, 56% of cases contained five or more LM and 44% were less than five LM. The dissected LM and matched myometrium were subjected to histologic evaluation to differentiate between LM and tumor-free myometrial tissues. Dissected tissue samples were snap-frozen for sequencing analysis. The general information of the selected cases for this study is summarized in Table 1. The dominant LM from each case was subjected to Sanger sequencing analysis for *MED12* mutation. Overall, 79% (23/29) of LM harbored *MED12* mutations (Table S1). This is consistent with the *MED12* mutation pattern found in most LM cohort studies.

### 3.2. Duplex Deep Sequencing of Myometrial Tissues

This study used Duplex deep sequencing technique (DDS) as it is more sensitive than traditional deep sequencing [22,23] by eliminating tens of thousands of erroneous mutations and capturing true point mutations on both DNA strands of the target genes (Figure S1B) [23]. Overall, the estimated error rate of DDS is <1 in 10 million [22], and this allows us to examine the low rate of mutations with extreme sensitivity and accuracy in myometrial tissue samples. When size-fractioned genomic DNA passed quality evaluation (Figure S1A), DNA libraries were prepared with adaptors specifically designed for DDS (Figure S1B). The target DNA components from coding regions exon 2 of *MED12* and exon 5 of *TP53* were then captured and subjected to DDS analysis. *TP53* exon 5 was used as a comparative internal DDS control.

**Figure 1:**
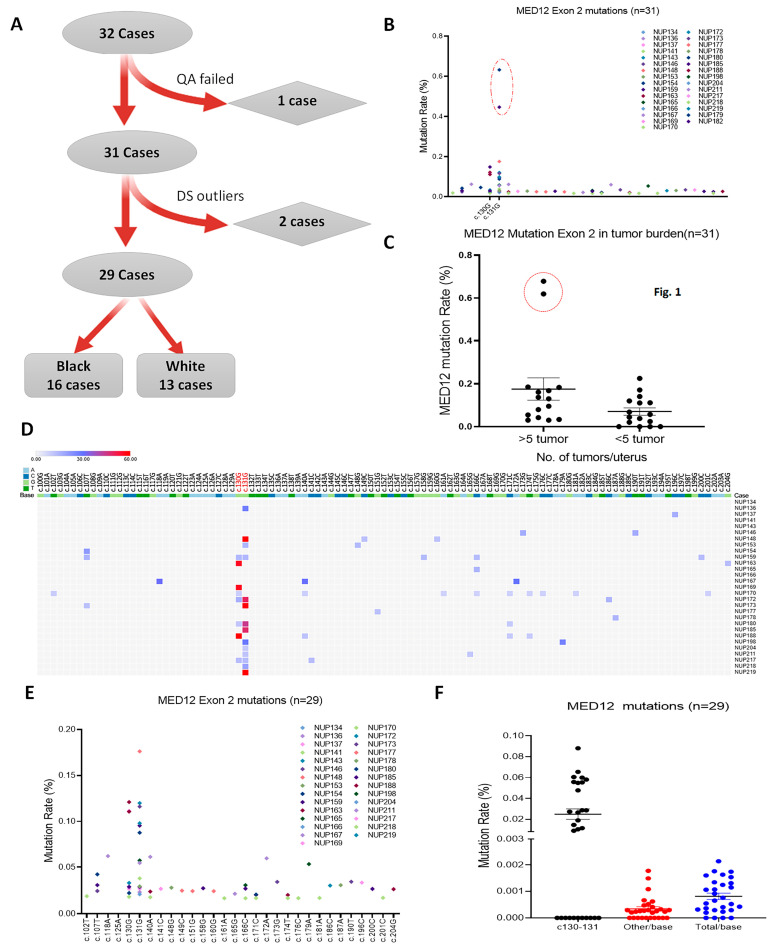
Point mutations of MED12 exon 2 detected by DDS in 29 cases. (**A**) A diagram illustrating the myometrial samples used for DDS. (**B**) Mutation plot showing the distribution of point mutations in exon 2 for 31 cases. Red circle highlights the 2 outliers. (**C**) Dot plot highlighting 2 outliers in the myometrium of 2 cases with >5 LM. (**D**) Mutation plot shows exon 2 mutation distribution in 29 cases by excluding 2 outliers. (**E**) Two-dimensional map illustrating the distribution of MED12 exon 2 mutations in each of 29 cases. Mutation frequency is marked from blue (low) to high (red). (**F**) Dot plot illustrating the MED12 mutations in c.130-131 and other sites.

After eliminating 1 sample that did not reach the passing scale of required sequence reads, 31 cases were subjected to the first round of DDS analysis (Figure 1A). Initial DDS mutation analysis revealed that two cases from myometrium had significantly higher mutation reads in comparison to the rest of the specimens (Figure 1B). The higher mutants from these two samples were from c.130-131 sites (Figure 1B). Further analysis revealed these two cases were obtained from myometrium with ≥5 LMs (Figure 1C). Since it is difficult to determine with absolute certainty whether these two were from potential LM contamination, to ensure the integrity of the DDS analysis, these two samples were eliminated from further analysis.

### 3.3. MED12 Mutation Analysis in Tumor-Free Myometrium

DDS covered DNA sequences in *MED12* exon 2 of nucleotides c.100 to c.204. The detected mutations had generally low frequency, with a mean of 0.81/10,000 reads/base in this region. Notably, there was a significantly different mutation distribution in exon 2, with the highest in c.130-131 (mean 1.24/10,000) and the lowest in other nucleotides (mean 0.34/10,000) (Figure 1F). As high as 62% of myometrial tissue (18/29) had detectable mutations at c.130-131 sites in the rate range of 0.18–1.76/1000 (Figure 1D). The mutation distributions in 29 cases across exon 2 were illustrated in Figure 1E. Apparently, c.130-131 enriched a high rate of mutations in tumor-free myometrium. Such a non-random distribution of specific nucleotide alterations was similar to our previous study of primary myometrial cells treated with chronic oxidative stress [8]. Findings strongly suggest that there is an unproportional high rate of *MED12* mutations in exon 2 code c.130-131 in myometrial cells.

### 3.4. MED12 Mutations in Association with Pathological Parameters

**Figure 2:**
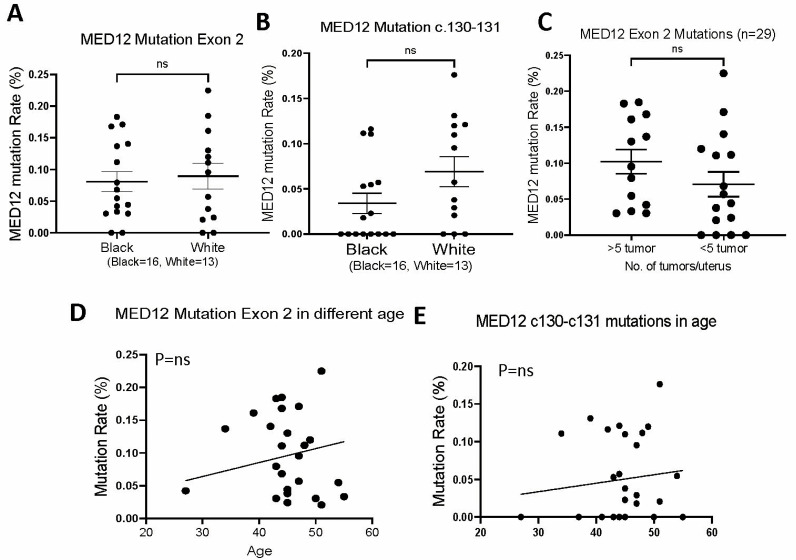
Mutation analysis of *MED12* exon 2 in association with race, number of tumors and age. (**A**,**B**) Mutation rate of *MED12* exon 2 (**A**) and c.130-131 (**B**) in myometrium of black (*n* = 16) and white (*n* = 13) women. (**C**) Mutation rate of MED12 exon 2 in myometrium with ≥5 and <5 LM. (**D**,**E**). Mutation rate of *MED12* exon 2 (**D**) and c.130-c.131 (**E**) in association with patient’s age. ns: no statistical significance.

To investigate the *MED12* mutation in association with the risk factors for LM development, we compared the mutation rate with several clinical and pathologic factors. *MED12* mutations of exon 2 and specifically c.130-131 in the myometrium of White and Black patients showed no significant difference (Figure 2A,B). Although there was a trend of higher *MED12* mutations in myometrium with ≥5 LM than with <5 LM, no statistical significance was reached (Figure 2C). The myometrium tissues were collected from patients with hysterectomies with ages ranging from 27 to 55 (Table 1 and Table S1). Mutations can be detected in all tissues of all ages (Figure 2D). When comparing the total mutations with age and c.130-131 with age, there was a slight increase in mutation rate in older age but did not reach statistical significance (*p* > 0.05, Figure 2E). The findings suggest that the mutation burdens in myometrial cells are similar in different races, ages, and numbers of tumors in this cohort of tissue samples.

### 3.5. MED12 Mutational Signature Analysis

**Figure 3:**
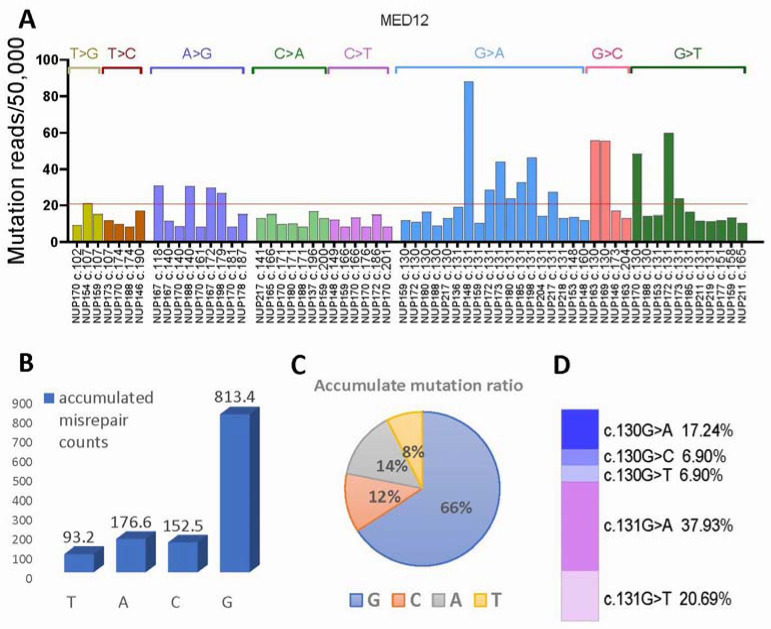
*MED12* exon 2 mutation signature analysis. (**A**) Distribution of mutation frequency in each of the 8 mutation signatures in 29 cases. (**B**,**C**) Accumulated point mutations and percentage in G, C, A, and T nucleotides. (**D**) Mutation rate of G > A, C, T in c.130-c.131.

The spectrum of base changes identified in myometrial cells of 29 cases was further analyzed for mutational signatures from the matrix of mutation counts across each tissue sample. Among 8 mutational signatures, 3 signatures of single base pair change were identified (Figure 3A). They were represented by multiple G > A, G > C, and G > T, with higher peaks at c.131 site, followed by c.130 and rarely at other sites (Figure 3A). Overall, mutations were highly enriched in G, accounting for 66%, followed by A (14%), C (12%), and T (8%) (Figure 3B,C). The mutational signature showed its unique set of peaks, which were similar but remained different from those of LM signatures [8]. The specific G transversion and conversion ratio are summarized in Figure 3D. The high mutation signatures of G presented in myometrial cells suggest that mutation selection was non-random among different nucleotides, and such a signature pattern may be highly relevant to oxidative stress-induced misrepair, previously identified in our in vitro study [8]. Furthermore, high mutations of G enriched in c.130-131 indicate the mutation selections in myometrial cells.

### 3.6. P53 Mutations in Exon 5 Detected by DDS

**Figure 4:**
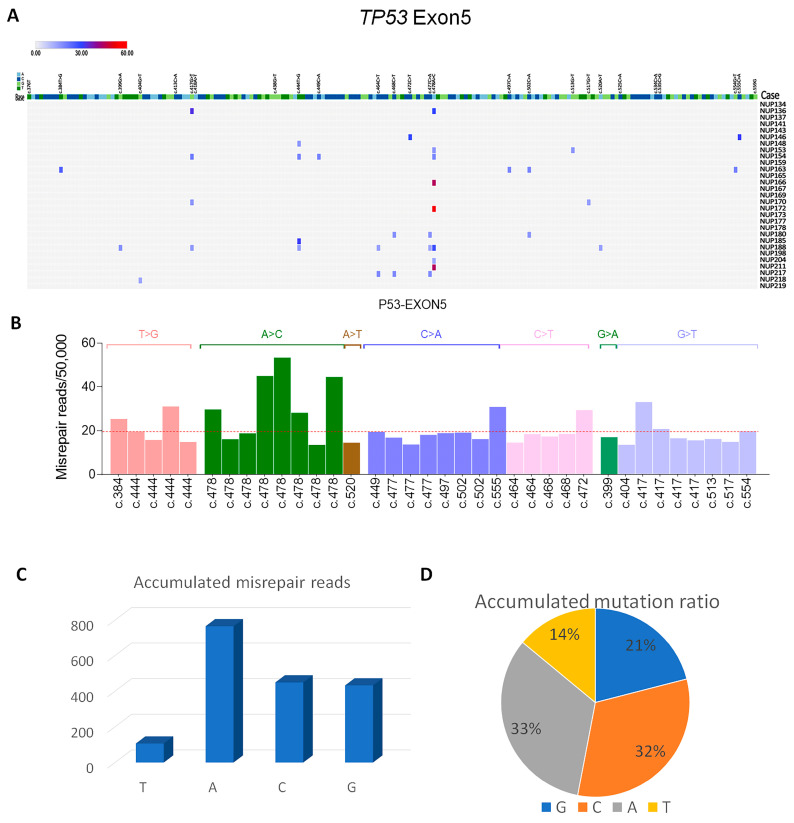
Mutation analysis of *TP53* exon 5 in 29 cases. (**A**) Two-dimensional distribution of base mutations in each of 29 cases. Mutation rate was indicated from low (blue) to high (red) bar. (**B**) 7 mutation signatures detected in *TP53* exon 5. (**C**,**D**) Accumulated point mutations and percentage in G, C, A, and T nucleotides in *TP53* exon 5.

The high rate of *MED12* exon 2 mutations found in myometrial cells was notable. To determine the general burden of single base misrepair or mutations in myometrial cells, we included *P53* exon 5 for mutation analysis by DDS. *P53* exon 5 harbors a high rate of mutations in Mullerian carcinoma in comparison to other exons [14,26] and it is thus a good target sequence for comparison purposes. As illustrated in Figure 4A, the rate of single nucleotide mutations in *TP53* was observed as a mean mutation rate of 0.57/100,000/base which was low. Notably, there were frequent somatic mutations enriched in c.477-c.478 and found in 11/29 cases of myometrium. There were 7 mutation signatures identified (Figure 4B). The dominant signatures were C > A and A > C enriched in c.477-c.478, accounting for over 65% of mutations (Figure 4C,D). Such dominant point mutations of A in *TP53* exon 5 and of G in *MED12* strongly suggest that despite the same genotoxic conditions, the types of mutations can vary for different genes.

## 4. Discussion

In this study, we demonstrated that *MED12* mutations occur in myometrial cells from hysterectomy specimens, giving insight into its role as a genetic driver of LM development. Our study showed there were detectable non-random *MED12* mutations in exon 2 enriched at c.130-131 nucleotides, in myometrial cells. This is the first study to uncover the somatic mutations in non-neoplastic myometrial cells of freshly collected tissue samples providing further insight into the mutational pressure in myometrial tissue.

The high rate leiomyomata in reproductive aged women harbor a high rate of *MED12* mutation. We hypothesize that there may be presence of *MED12* mutations in some non-neoplasmic myometrial cells. However, detection of *MED12* mutations in normal myometrial tissue is technically challenging. This is largely due to the fact that (1) standard next-generation sequencing NGS has low sensitivity in detections of low rate of mutations [19] and (2) the estimated error rate in standard NGS is 10^−2^ to 10^−3^/per base [22]. These errors can be induced by PCR amplification misincorporation, tissue-processing stress, DNA damage during extraction, and probe hybridization and capture errors. The low rate of sequencing errors from general NGS is acceptable for some research purposes or molecular analyses, but it has a major limitation in conditions that require greater accuracy to detect extremely low-frequency mutation, such as searching for true mutations in normal myometrial cells in this study [27].

Duplex deep sequencing (DDS) is a new platform for next-generation sequencing (NGS). A DNA library is prepared by adding barcoded tags in both stranded DNA which will facilitate in mutation detection with higher accuracy and lower error rates. The degenerate barcoded tags in each strand will aid in identification of reads originating from each DNA strand. Since the two strands are complementary, true mutations that are found at the same location on both strands were recorded. Therefore, any errors or mismatch in one strand can be eliminated. Studies showed that DDS can detect mutations at frequencies as low as 5 × 10^−8^, which is more than 10,000-fold specificity in comparison to traditional next-generation sequencing methods [22]. Therefore, we used the DDS technique in this study and were able to reliably detect and calculate the *MED12* mutations in normal myometrial cells.

LMs are the monoclonal smooth muscle tumor which are derived from single myometrial cell. The causes of neoplastic transformation from myometrial cells are largely unknown. It warrants further investigation into the biology and molecular nature of myometrial cells and this will aid our understanding of the high burden of LM in reproductive-aged women. The human myometrium is organized by an intricate network with terminally differentiated mullerian smooth muscle linages and with rich extracellular matrix including connective tissues of vessels, fibroblasts, and niche of inflammatory cell network [28]. In response to tissue regeneration, myometrium can adapt to dynamic changes, such as expansion/hypertrophy during puberty, cell proliferation or apoptosis during cyclic hormonal change, and finally in involution at postpartum [29].

The causes for mutations in non-neoplastic myometrial cells remain unknown. The patterns of *MED12* mutations found in myometrial cells were similar to those found in primary myometrial cells treated with oxidative stress as reported in our previous study [8], suggesting that ROS may be one of the critical local microenvironmental factors promoting *MED12* mutations. Specifically, the high single G and C transversion/conversion we observed for *MED12* mutational signature in myometrial cells are commonly seen in oxidative-related carcinogenesis [13,14]. Oxidative stress is common in functional uterus and is tightly regulated by the local microenvironment [9,10,11,12]. The functional myometrium in reproductive-aged women encounters the cyclic contractions which promote local hypoxia [30], and increased inflammation during the menstrual cycle and menses [31]. Oxidative burden can be further exacerbated by lifestyle factors such as diet, physical activity, and psychosocial variables. This higher oxidative burden in the myometrium can result in oxidative DNA damage and misrepair [32]. This may partially explain the increased MED12 and p53 mutations in tumor-free myometrial cells. However, the rate of mutations remains to be quite low, as 40% of myometrial tissue samples did not detect *MED12* mutations with our cutoff of 50,000 cell detection levels.

Our findings of MED12 mutations in normal myometrial cells suggest that ROS burden may be a major risk factor for *MED12* mutation [8,32]. This may be supported by a finding of age-matched myometrial tissues from women with LM have higher immunoreactivity for 8-OHdG compared to the myometrial tissues without LM [8]. The expression of proteins associated with oxidative DNA damage and repair, were higher in LM and matched myometrium [32]. Along with increased oxidative stress in the myometrium, there are increases in DNA damage through 8-OHdG formation and misrepair. Interestingly, the *P53* mutational signatures were different from *MED12* in this current study. *P53* harbors more mutations in A and C in contrast to G found in *MED12*, indicating mutation selection to certain genes. The exact molecular mechanism remains unknown.

LMs are monoclonal tumors from myometrium. Study showed that early-life with adverse environmental exposures can impose a risk for LM development. The uterine smooth muscle cells are the target for developmental programming during early-life hormone exposure [33,34]. Many environmental factors, different ethnic backgrounds and lifestyle can also impose a high oxidative stress in the myometrium [35,36,37,38]. Due to the small sample size of this study, we did not observe a significant difference of *MED12* exon 2 mutations in myometrium of different races, and also no association with tumor burden or patients’ age.

To date, data on the molecular pathogenesis of LM remain insufficient to elucidate the reasons for the high incidence of LM burden. Detection of the single-cell gene mutation burden by DDS allows us to explore the early stages of *MED12* mutations in non-neoplastic myometrium and may significantly improve our understanding of the high incidence of LM in the uterus. The discovery of the G/C rich mutational signatures further supports the potential carcinogenic role of ROS in promoting *MED12* mutations. Notably, our observation of non-random mutational signatures and target hot spot mutations in *P53* and *MED12* are indicative of different mutation selections for different genes, for cell survival and tumor development. Limitations of this study include the small sample size, lack of mutation analysis in other exons of *MED12*, and technical challenges in examining different anatomic sites of myometrial tissues. For the first time, we have demonstrated similar mutations in exon 2 of the *MED12* gene found in LM, in myometrial cells, strongly implicating the *MED12* mutation, specifically at c.130-c.131 as an early driver of LM.

## 5. Conclusions

This study is the first to report MED12 mutations in non-neoplasm myometrial cells. Our findings will help to understand the molecular basis for a high incidence of uterine leiomyoma in women of reproductive age.

## Figures and Tables

**Table 1:** General information about the cases used in this study.

	Black	White	Total
No. of Cases	16	13	29
Age	43.12 ± 1.79	45.54 ± 0.97	44.31 ± 1.08
>5	56.25% (9/16)	30.77% (4/13)	44.83% (13/29)
<5	43.75% (7/16)	69.23% (9/13)	55.17% (16/29)
MED12 Mut in LM	87.50% (14/16)	69.23% (9/13)	79.31% (23/29)
MED12 Mut in MM	50.00% (8/16)	76.92% (10/13)	62.07% (18/29)

## Data Availability

All data are available upon request.

## Supplementary Materials

The following supporting information can be downloaded at: https://www.mdpi.com/article/10.3390/cells13171432/s1, Figure S1: A sketch diagram of duplex deep sequencing analysis; Table S1: Case details for clinical and molecular findings.
