# Supplementary material for: Somatic MED12 Mutations in Myometrial Cells
Source: Cells. 2024 Aug 27;13(17):1432. doi: 10.3390/cells13171432 (PMC11394142; doi:10.3390/cells13171432)
Supplement: Supplementary file 1 [file cells-13-01432-s001.zip › cells-3133120-supplementary.pdf]

Supplementary Table S1 Case details for clinical and molecular findings

	Case	RACE	AGE	Number	MED12 Muts in dominant LM	130-131 Muts in MM(/50,000)
case 1	NUP134	AA	37	<5	NO	0
case 2	NUP136	AA	54	>5	c.131G>A	27.29
case 3	NUP137	AA	55	>5	c.130G>C	0
case 4	NUP141	AA	37	<5	c.130G>T	0
case 5	NUP143	WT	41	<5	c.131G>A	0
case 6	NUP146	AA	44	<5	c.130G>C	0
case 7	NUP148	WT	51	<5	c.131G>A	88.12
case 8	NUP153	WT	47	<5	c.107T>G	14.55
case 9	NUP154	AA	27	>5	c.130G>C	0
case 10	NUP159	AA	44	>5	c.126-131DEL6	28.57
case 11	NUP163	AA	34	>5	c.130G>C	55.34
case 12	NUP165	AA	50	>5	c.130G>A	0
case 13	NUP166	WT	44	<5	c.130G>C	0
case 14	NUP167	AA	43	>5	c.131G>T	0
case 15	NUP169	AA	48	<5	NO	55.78
case 16	NUP170	AA	47	<5	c.131G>A	9.01
case 17	NUP172	WT	39	>5	c.131G>T	65.48
case 18	NUP173	AA	42	<5	c.131G>A	58.11
case 19	NUP177	WT	45	<5	NO	0
case 20	NUP178	AA	43	>5	c.131G>A	0
case 21	NUP180	WT	45	>5	c.131G>A	54.86
case 22	NUP185	WT	47	>5	NO	47.71

case 23	NUP188	WT	44	>5	c.131G>T	60.53
case 24	NUP198	WT	44	<5	NO	28.65
case 25	NUP204	WT	51	<5	c.131G>A	10.36
case 26	NUP211	AA	45	<5	c.131G>A	11.49
case 27	NUP217	AA	43	>5	c.131G>A	13.25
case 28	NUP218	WT	45	<5	NO	18.93
case 29	NUP219	WT	49	<5	c.130G>C	59.95

Supplementary Figure S1

A

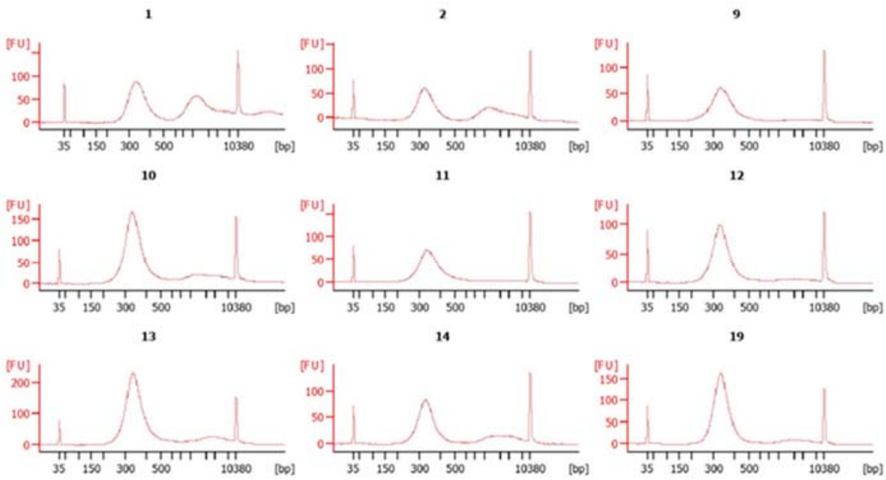

B

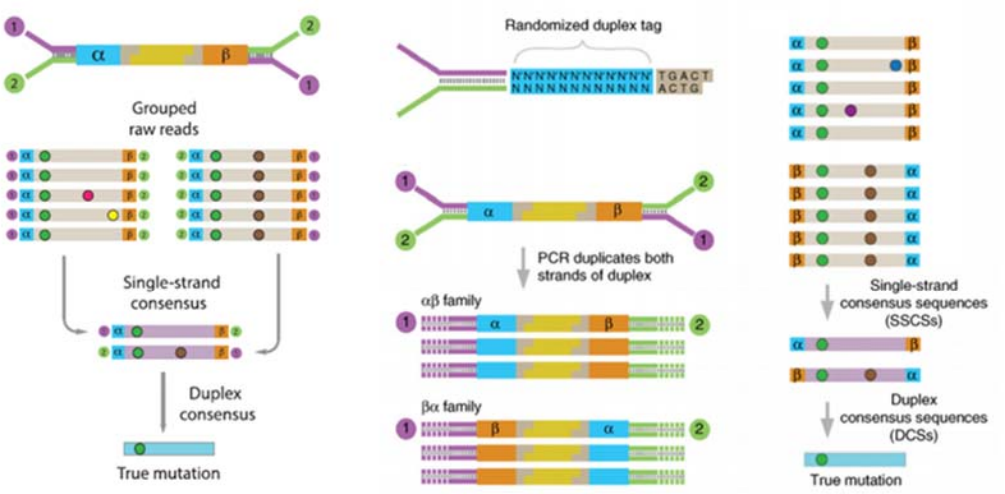

Nat Protoc. 2014 Nov;9(11):2586-606.
